# Transforming maternal health in Ethiopia: Leveraging human-centered design to co-create innovative behavioral interventions

**DOI:** 10.1371/journal.pgph.0006021

**Published:** 2026-02-24

**Authors:** Yihunie Lakew, Biruk Melaku Ayalew, Habtamu Tamene, Simon Heliso Kuka, Tewabech Tesfalegn, Danielle Piccinini Black, Bee-Ah Kang, Rajiv N. Rimal

**Affiliations:** 1 Center for Communication Programs, Johns Hopkins University, Addis Ababa, Ethiopia; 2 Center for Communication Programs, Johns Hopkins University, Baltimore, United States of America; 3 Carey Business School, Johns Hopkins University, Baltimore, United States of America; 4 Department of Health, Behavior and Society, Johns Hopkins University Bloomberg School of Public Health, Baltimore, United States of America; PLOS: Public Library of Science, UNITED STATES OF AMERICA

## Abstract

A significant number of maternal and child births in Ethiopia are at elevated risk because they occur at home and/or because of low rates of antenatal care visits. This study applied a human-centered design (HCD) approach to develop contextually appropriate solutions that can promote institutional delivery and a greater number of antenatal care visits. The HCD approach (a five-step design-thinking process) was conducted in two rounds of workshops. The first-round included pregnant women living with vulnerabilities and their husbands, while the second round engaged healthcare providers and community influencers. The second round of workshops were anchored on findings from the first round. Facilitators trained in the HCD conducted these workshops. A total of 204 participants —pregnant women, husbands, healthcare providers and community influencers, organized into 16 design teams, participated in the workshops and prototype testing. Prototypes were tested and iterated to ensure feasibility and acceptability. The design teams produced stakeholder maps, personas, journey maps, and affinity clusters to deepen their understanding of the challenges and foster stakeholder empathy. Key insights harvested from both round of workshops guided the refinement of the design challenges, resulting in the formulation of twelve “How might we…” statements to facilitate ideation. The resulting prototypes —a self-paced audio program interventions featuring real-life stories and visual print materials—were implemented over four months in two Oromia districts, with Women Development Armies playing a pivotal role in facilitating the rotation of home-based audio interventions. This participatory and iterative process, guided by design thinking, emphasizes the potential of human-centered design to develop contextually appropriate and feasible solutions aimed at enhancing maternal health service utilization in resource-constrained settings.

## Introduction

The United Nations has set the goal of reducing maternal mortality ratio by 2030 to below 70 per 100,000 live births, neonatal mortality to lower than 12 deaths per 1,000 live births, and under-5 mortality to at least as low as 25 deaths per 1,000 live births [1]. Despite a 38% improvement in maternal and child mortality rates between 2000 and 2017, progress remains uneven, with sub-Saharan Africa bearing a disproportionate burden [2]. Similarly, Ethiopia’s maternal mortality ratio dropped by 39% from 676 per 100,000 live births in 2011 to 412 in 2016 [2,3], and a reduced estimates of 244 per 100,000 livebirths in 2023 [4], however, it remains high and ranks among the top ten countries [5].

The Ethiopian health system is organized into a three-tiered structure prioritizing primary service coverage and the enhancement of health outcomes. The first tier comprises primary healthcare units (PHCUs)—including health centers and about five health posts—that serve populations of up to 25,000 individuals. The second tier consists of district hospitals capable of serving populations of up to 1.5 million. The tertiary level encompasses specialized hospitals designed to provide advanced care for approximately five million residents [6]. Each health post is staffed by two Health Extension Workers (HEWs) and is supported by volunteer community health workers known as Women Development Armies (WDAs).

The national antenatal care (ANC) guidelines recommend pregnant women initiate care within the first 12 weeks at health centers, with up to eight contacts at nearby health posts or centers, concluding again at the health center. However, only 43% of Ethiopian women had attended at least four antenatal care visits during their last pregnancy [7]. The 2019 Ethiopian Demographic and Health Survey indicates that, although skilled birth attendance has risen from 28% in 2016 to 48% in 2019, many women still deliver at home without professional support [8]. Most births are attended by unskilled family members at home, increasing risks of complications like severe bleeding, infections, and sepsis [7].

Expanding access to antenatal care and institutional delivery services is essential to reducing maternal and neonatal mortality, as facility-based deliveries provide a safer environment and reduce post-partum complications. Studies suggest that institutional delivery could lower maternal deaths by 16–33% [9]. Several studies indicate that attending antenatal care is a major gateway behavior to institutional delivery as women who have completed the recommended antenatal care visits in health facilities are more likely to deliver in health facilities [10–12]. Use of antenatal care services and their health outcomes are largely shaped by factors that affect both the decision to seek and the ability to use these health services. These factors include complex underlying social and structural factors, such as distance, [13–16] low income, [13,17] lack of transport and cost, [17] low literacy, [14,16] and inadequate media exposure [15]. In addition, studies show that couple communication and joint decision making significantly improves maternal service utilization [18,19]. These factors contribute to the vulnerability of households and individual woman, limiting their access to essential maternal and child health services and exacerbating the risk of maternal mortality [20].

Human-Centered Design (HCD) is an important design thinking framework for many different problem-solving that are empathy-centric. Design thinking is one of those empathy-centric problem-solving processes and was the one we operationalized on our project. It is an iterative, participatory approach that adopts user-centered solutions for promoting behavior change [21], centering stakeholders’ values (engaging both end-users and other key stakeholders), and has demonstrated success in improving healthcare accessibility and outcomes [22–24]. Research suggests that facilitators and participants in the design thinking processes feel empowered and appreciate the collaborative spirit, [25] thereby enhancing the usability and sustainability of solutions. It uses group of methods and principles that support gaining insights and applying learnings about human beings and their interactions with the environment to design innovative solutions that meet their needs and aspirations [21,23].

In Ethiopia, there is a lack of empathy-centric interventions to improve antenatal care and institutional delivery services. Our main design challenges of focus were reimagining how to support pregnant women to 1) attend antenatal care during their first trimester and 2) deliver in a health facility. We applied an HCD approach, adopting a design thinking framework to address these design challenges and understand the needs, capabilities, and limitations of different stakeholders including end users. This included intentionally engaging vulnerable pregnant women, their families, providers, and other key stakeholders related to the design challenges throughout our design thinking process. In this paper, we describe the HCD process we undertook to generate solutions that can promote institutional delivery and a greater number of antenatal care visits among pregnant women in Shalla and Siraro Woredas. Besides this, a separate paper investigating the outcomes of this approach will be forthcoming. While our overall research program seeks to develop, implement, and evaluate the effectiveness of HCD informed solutions to improve ANC and institutional delivery, this paper focuses explicitly on the HCD development process and application, which involves iteratively reviewing the literature and refining the design solutions that emerge from our engagement with stakeholders.

## Methods

### Design setting

Ethiopia is subdivided into 12 regional states and two administrative cities and regions, which are further partitioned into zones, woredas, and then kebeles (the lowest administrative unit). The estimated total population of Ethiopia is projected to reach approximately 109 million, with a total fertility rate of 4.1 births per woman. Within this context, Oromia Region accounts for a population of around 41 million residents, including Shalla Woreda with a population of 222,843 and Siraro Woreda with 214,746 individuals [26]. In the study areas, there are a total of 15 health centers—comprising eight in Shalla and seven in Siraro—that currently provide maternal health services alongside other healthcare provisions. The implementation research as well as the design thinking work was conducted in two districts (Shalla and Siraro woredas) in the Oromia Regional State, the most populous regional state in the country.

### Ethical approval

The first phase of the study was reviewed and approved by the Johns Hopkins Bloomberg School of Public Health Institutional Review Board (IRB00024473) on July 18, 2023, and the approval was extended to Phase 2 on July 16, 2024. In order to conduct HCD processes, we also obtained ethical approval for public health practice by the Johns Hopkins Bloomberg School of Public Health Institutional Review Board (IRB00023366) on January 4, 2023. This implementation research was approved in October 2023 by the Ethiopian Public Health Institute (EPHI), a governmental public health institution located in Addis Ababa, Ethiopia (EPHI-IRB-510–2023).

Verbal consent was employed throughout our HCD process due to the involvement of low-literacy levels and cultural sensitivities around written signatures and the presence of witnesses. Given the minimal risk posed by the HCD activities and the absence of sensitive subject matter, verbal consent was deemed appropriate and ethically sufficient. A clear and concise consent script was developed and approved by the Institutional Review Board (IRB). The script included all required elements of informed consent: the purpose of the study, voluntary nature of participation, procedures involved, expected duration, potential risks and benefits, confidentiality safeguards, the right to withdraw at any time, and relevant contact information. Facilitators confirmed participants’ understanding of this information prior to obtaining consent by asking a direct question, such as, “Do you voluntarily agree to participate in this study?” Participation proceeded only upon receipt of an affirmative response. Facilitators documented consent in accordance with IRB protocol, and information sheets were provided when appropriate. All consent records were securely stored. All procedures carried out in accordance with the study approved protocol.

This research initiative did not offer direct financial incentives to workshop participants. However, participants received reimbursement for transportation expenses and a daily allowance to cover meals and incidental costs incurred during their participation.

Health Extension Workers (HEWs) and Health Development Agents (HDAs) participated in two review meetings focused on performance assessment, challenges, and lessons learned, during which they received training and engaged in review activities. The project covered per diems, accommodation, and travel expenses for these meetings, which also included stakeholders such as Primary Health Care Unit (PHCU) directors, Woreda Health Office officials, and community representatives.

### Design sprints in two workshop rounds

A two-round series of co-design workshops were held between April 2023 and February 2024 to inform maternal health initiatives through community-driven insights. The first-round of workshops with 4 concurrent separate sessions was conducted from 30 October to 28 November 2023 that involved pregnant women and their husbands to enable open and safe dialogue. These discussions captured lived experiences, key challenges, and priorities to guide the human-centered intervention design. A three-week intersession period allowed the facilitation team to synthesize findings to inform the second round, conducted from 10 January to 25 February 2024. This phase brought together key health and community stakeholders, including Health Extension Workers, midwives and nurses, Primary Health Care Unit (PHCU) leadership, Woreda Maternal and Child Health (MCH) coordinators, Women’s Health Development Army (WHDA) members, and influential community leaders, such as religious figures, Kebele administrators, and women’s affairs coordinators. The two rounds of workshops, each spanning three days, aimed to translate community insights into actionable, locally relevant strategies (solutions) through a participatory design process.

### Design teams

Our comprehensive design thinking framework and methodology were orchestrated by a dedicated core team (including HCD experts and other relevant program staff, researchers and workshop facilitators). The workshop facilitators were selected and trained based on their local language skills to ensure effective communication and inclusiveness with participants from community members. The core team played a major role in providing overall guidance of the HCD process, preparing HCD tools, selecting stakeholders, planning and organizing workshops, synthesizing outputs and translation of the prioritized ideas into workable prototypes and testing them. The core team initially developed a Bullseye Diagram to decide which stakeholders get involved in the design workshops. In the context of selecting stakeholders for the initial workshop, the team highlighted concerns regarding potential power imbalances that may arise from convening all stakeholders simultaneously. Specifically, there is a risk that the perspectives of marginalized rural women and men could be overshadowed by healthcare providers. As a result, the core team decided to initially conduct workshops with homogenous groups with pregnant women and husbands separately. The core team subsequently engaged additional stakeholders through concurrent workshops, aiming to incorporate and build upon the perspectives and insights provided by pregnant women and husband groups. The demographic profiles of design team participants in two workshop rounds are indicated in (**S1 Table**).

**Fig 1:**
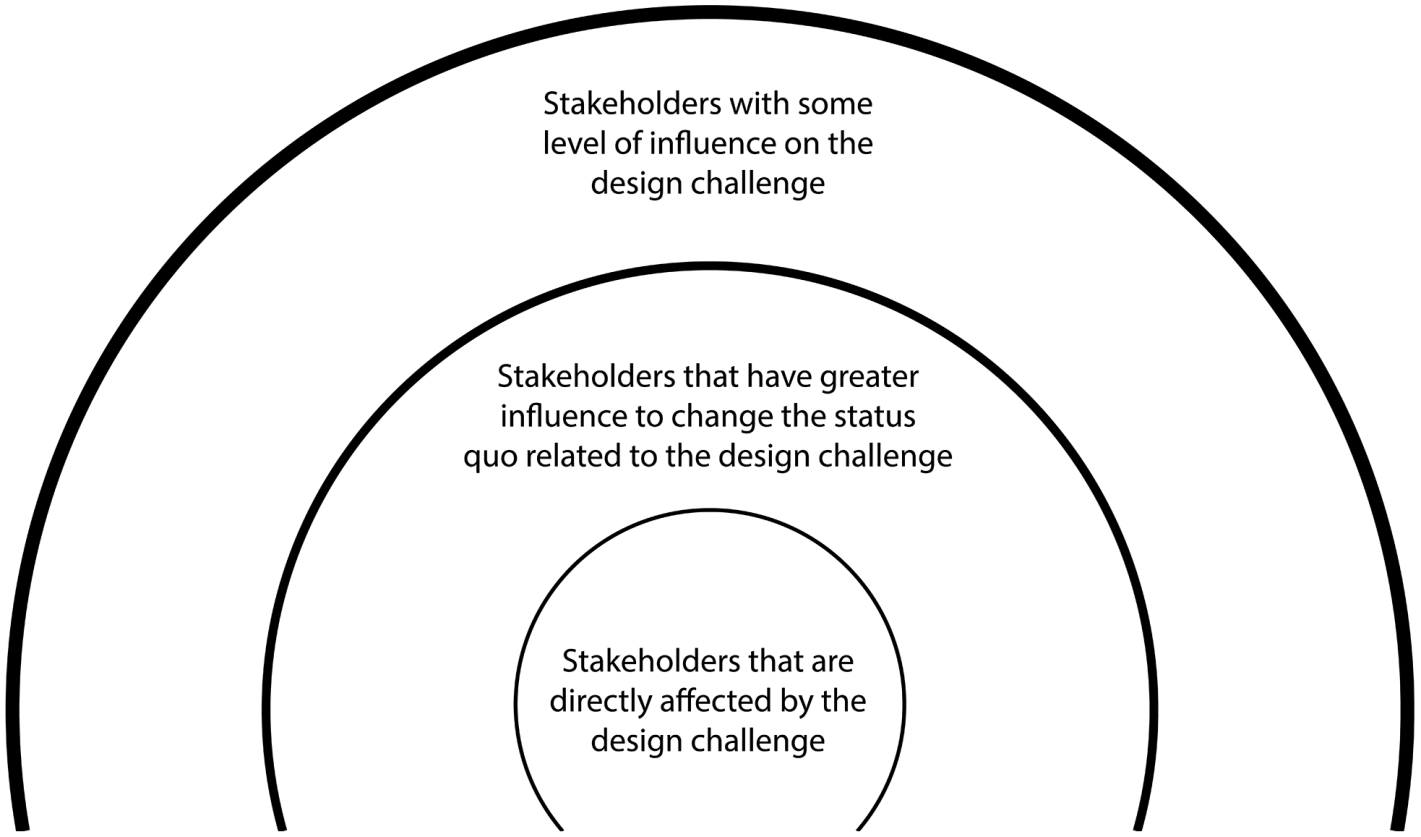
The Bulls Eye Diagram – a template used to map stakeholders in the design workshops.

In both round of workshops, a total of 16 design teams from pregnant women, husbands, and healthcare providers and community influencers were organized and engaged. Design teams in each workshop used pre-designed templates, for example, the Bulls Eye Diagram template used to map stakeholders in these workshops to categorize as critical, important, or peripheral stakeholders (**Fig 1**).

### Recruitment and selection of workshop participants

The core team initially created a Bullseye Diagram to determine the stakeholders to be involved in the design workshops. Utilizing this diagram, the team identified participants for both rounds of workshops. Then, participants in the first-round workshops (pregnant women and husbands) were selected based on pre-defined vulnerability criteria. These recruitment criteria were identified based on a comprehensive literature review and analysis of the 2016 EDHS data: 1) Long distance from residence to health facilities (more than 30-minute walk), 2) Inability to read and write, 3) Poor housing conditions (house roof with corrugated sheet or tukul type), 4) Lack of access to a mobile phone, 5) Large family size (4 or more children), and 6) Limited land ownership (less than 2-timad land per household). The project staff worked with HEWs to identify and recruit pregnant women and husbands who met at least three of the criteria mentioned above. For this round of workshops, we first visited 201 households with pregnant women, using local guides, to identify those who are eligible and ultimately found 96 who were eligible and willing to participate. We stopped our house-to-house visits once we reached 96 because that was the number we needed. None of the eligible women and men we invited declined.

The stakeholders who participated in the second-round workshops were frontline health workers and actors. In these workshops frontline health care providers (HCPs) who promote and deliver essential health services directly to the community with the greatest access to clients and patients were included [27]. The selection of these stakeholders was through a stakeholder mapping exercise based on the following considerations: Living in the implementation woredas for at least six months, ability to speak in the local language and familiarity with the norms and culture of the community. These considerations were made because we wanted to make sure they could meaningfully contribute to the design process in empathizing and creating a solution that works for the context. Twenty-four eligible stakeholders were invited and willing to participate in the second-round workshops (no decline).

### Operational definitions

(S2 Table)

### Design thinking process

For our design thinking work, we adopted the Design Thinking (DT) framework developed by Stanford d.school [28]. It is an iterative process—with the five steps distinct, yet interconnected, steps of Empathize, Define, Ideate, Prototype, and Test—allowed for a structured and flexible framework to co-create solutions with end users and other stakeholders. The iterative nature of design thinking encourages the exploration of ideas in an organic way; not being tied to a linear process but rather allowing the insights that emerge to dictate how to flow through it to ultimately get to the final solution(s).

Two differentiating factors of design thinking, when compared to other problem-solving methodologies, are its iterative nature and divergent and convergent processing. The approach alternates between divergence and convergence to deeply openly question and explore (divergence) and make sense of and refine ideas, insights, and ultimately solutions (convergence). The Empathy phase diverges as it seeks to capture a wide range of perspectives and environmental factors impacting the community by leveraging both primary and secondary research, with a strong emphasis on primary—learning directly from and designing with key stakeholders (pregnant women and husbands- end users). In the Define phase, divergence continues as we learn about the landscape of the design challenge of focus, placing the stakeholders we identified in the empathize phase at the center of insights related to the structural, regulatory, environmental, and cultural factors. At the end of the Define phase, we find the first point of convergence, by narrowing in on the core aspects of the design challenge to identify the problem’s critical dimensions. The Ideation phase diverges once again, encouraging exploration of various ideas and solutions related to the design challenges, before converging to prioritizing the most promising ones. This convergence continues in the Prototype phase, where a specific solution is developed. Finally, the Testing phase diverges by gathering open-ended feedback to further refine the intervention based on community input.

**Fig 2:**
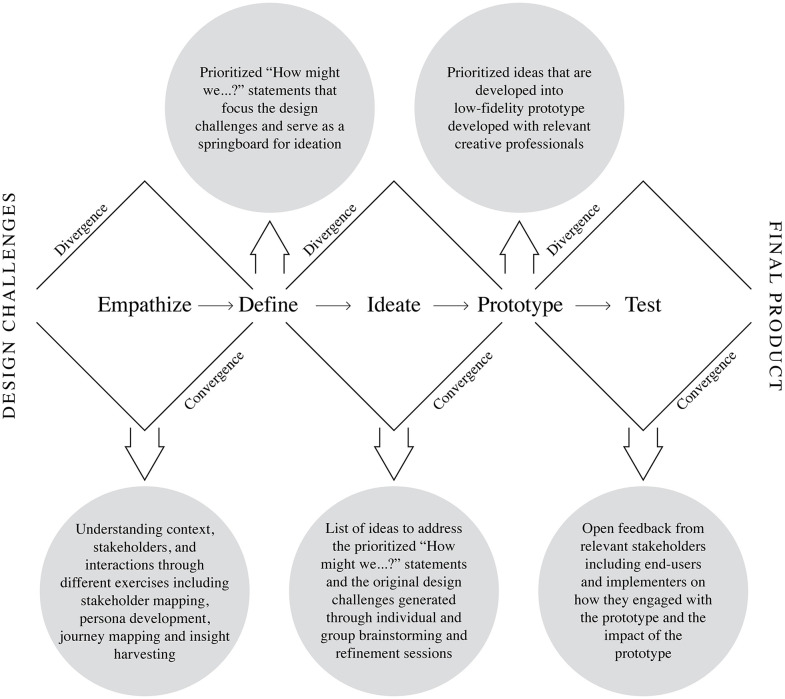
Divergent and convergent phases with five steps applied during our HCD process.

In the context of this design thinking work, our process commenced with the formulation of design challenges derived from an initial comprehensive desk review. This review focused on identifying barriers and facilitators related to antenatal care and institutional delivery, thereby establishing a foundational understanding for subsequent design activities. The divergent and convergent phases with five steps of the design thinking process are shown in (**Fig 2**).

Our design-thinking process was implemented in two rounds of workshops. The initial set of workshops were with teams of pregnant women and husbands, as the primary stakeholders identified in the initial stakeholder mapping exercise. These two stakeholder groups were treated separately in different workshops to ensure a safe space for them meaningfully to contribute to the process. This included 16 different sets of workshops (8 teams with pregnant women and 8 teams with husbands) comprising in total 16 design teams (eight in each woreda). Half of these workshops were only with eligible pregnant women, and the other half only with eligible husbands. Each workshop had six participants. This makes the total number of participants 96 (16 *6).

Subsequently, the second-round of workshop with healthcare providers (HCPs) and other community influencers were conducted in the same two woredas, as this stakeholder group was identified during the stakeholder mapping as critical to the design challenges of focus. They were organized into eight different teams, four focusing on the first design challenge and four on the second. This round of workshops was designed to build on the outputs from the first round to ensure key insights and ideas that emerged from pregnant women and husbands were not lost. The workshops were organized with a total of 48 participants (healthcare providers and other community influencers).

In both workshops, participants completed three Design Thinking steps—Empathizing, Defining, and Ideation. The core team ensured continuity by presenting outputs from the first workshop (stakeholder mappings, personas, journey maps, insights, how-might-we statements and initial ideas). The second workshop largely confirmed first-round insights, reducing the need for re-validation. Ideation involved rapid individual brainstorming, group brainstorming, and prioritization. Facilitators integrated ideas from both rounds before group discussion.

**Fig 3:**
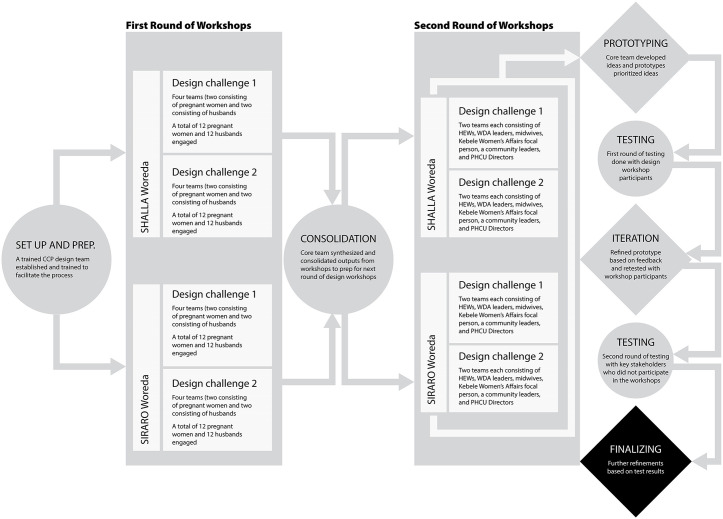
Summary of the Design Thinking process as applied to co-designing prototype solutions.

Overall, a total of 204 pregnant women and their husbands and healthcare providers and local community influencers were engaged as participants in the design workshops and prototype testing. The overall design thinking process employed in this study to develop solutions aimed at enhancing antenatal care and institutional delivery among vulnerable women is illustrated in (**Fig 3**).

### First round workshop activities with pregnant women and husbands

#### Empathy phase.

The first round of workshops was conducted among teams of pregnant women and husbands, held separately to provide a safe and supportive environment. Participants in each team (group) were guided through empathy phase activities, including stakeholder mapping, persona development, and journey mapping. In the stakeholder mapping exercise considered the design challenge of focus, participants identified key individuals and groups influencing their access to health services, categorizing them as critical, important, or peripheral stakeholders using a stakeholder mapping templet indicated above in Fig 1.

**Fig 4:**
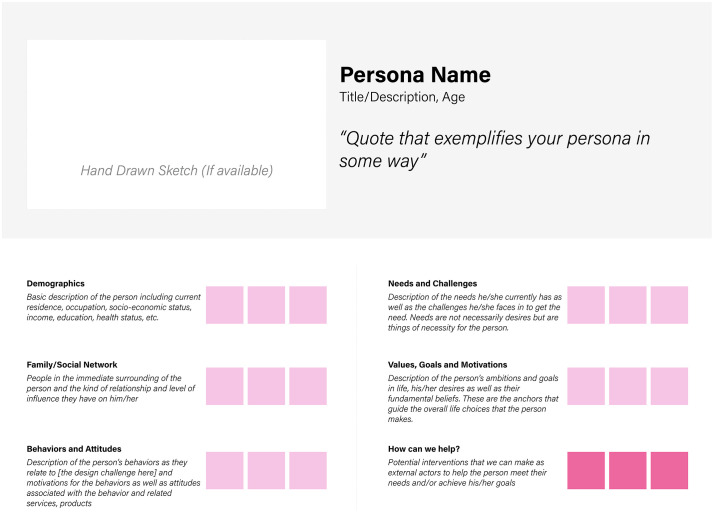
Persona development template used in design workshops.

Participants then created personas representing typical pregnant women and husbands within their communities which highlighted the demographics, family/social networks, behaviors and attitudes, needs and challenges, values, goals and motivations, and sources of information. These personas were followed by journey maps, which outlined the typical experiences of the pregnant women personas from early pregnancy to childbirth highlighting the different steps of their journey and the related feelings, challenges and opportunities. Personas were summarized using a template (**Fig 4**).

**Fig 5:**
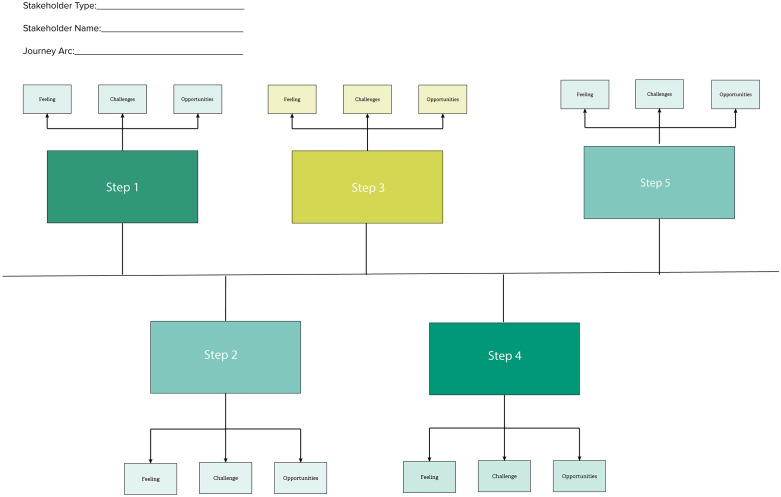
Journey mapping template used in design workshops.

Two journey arcs were developed: one tracing the journey from pregnancy suspicion to five months of gestation, and the other from preparing for delivery through childbirth. Journey mapping exercises were done using the journey map template (**Fig 5**).

### Define phase

#### Affinity clustering and insight harvesting:.

After completing the empathy phase activities, the workshop participants dive into the define phase, through a modified rose, bud, thorn exercise, discussing, analyzing and synthesizing information about the design challenge landscape. To support this process, between workshop sessions, facilitators synthesized lists of positives and negatives generated by the workshop participants using an affinity clustering exercise, grouping related factors to generate insights. These synthesized clusters were presented to participants at the beginning of the final day of the three-day workshop to validate their accuracy and relevance. Using the insights from these clusters, facilitators guided participants to define core problems by developing “How Might We” statements rooted in all of the information that emerged during the workshop, specifically during the empathize and define phase activities. These statements reframed the challenges identified and provided a springboard for ideation.

### Initial ideations

In the first round of workshops, the ideation step was done in three sub steps: rapid individual brainstorming, followed by group brainstorming, and then prioritization of ideas. Participants were encouraged to generate ideas addressing all the how-might-we statements that they have prioritized. Workshop facilitators played a significant role in using specific techniques that allowed participants who could not read and write to contribute. Ideas communicated verbally by participants were captured on pieces of colored paper by the facilitators.

### Second round workshop activities with HCPs and additional stakeholders

A second round of workshops was held with frontline health workers and local actors. Outputs from the first workshop were presented to these new participants, who built upon the existing personas and journey maps, and created new ones specific to healthcare providers and community influencers.

Participants in this second round underwent a similar process using similar templates as were used in the first round of workshops (stakeholder mapping, personas, journey maps, as well as define and ideation). Then they developed stakeholder mapping, personas, journey maps, positives and negatives based on their own experiences and conducted affinity clustering exercises to identify insights themselves. Additional how-might-we statements were generated from this round, with all statements from both rounds combined and jumbled to ensure that insight formulation was unbiased. This led to a set of prioritized how-might-we statements addressing critical barriers and opportunities for antenatal care and institutional delivery.

### Insights and how-might-we statements (HMWs)

The design thinking process revealed several critical insights into challenges faced by vulnerable pregnant women and their communities in accessing maternal health services. These insights informed the design process and led to the development of specific how-might-we statements that guided ideation. The HMWs were formulated under define phase, based on insights harvested during two rounds of workshops. We built in ‘yes… and’ exercises in the second round of workshop, whereby participants were presented with insights and ‘How might we…’ statements that pregnant women and husbands identified and then affirmed these statements before adding on additional insights and statements. To a large part, participants in the second round of workshop agreed with the insights and statements that came from the first round of workshop, which eliminated the need for us to go back to the first workshop participants for validation. A total of 24 HMWs were identified from the first and second round workshops. After overlapping HMWs reduced, twelve such statements were identified for the second-round workshops ideation. The most notable insights and how-might we statements are indicated below.

**Table:** 

Insights	How-might-we statements
*Limited Joint Decision-Making in Utilizing Maternal Health Services:* Maternal health is always left to women. Couples often lack joint decision-making regarding maternal and child health, particularly around ANC and institutional delivery. Husbands are frequently uninformed about their role or what to do when their wives approach delivery. Consequently, pregnant women may delay leaving home, fearing an extended absence from household duties. This delay often results in spontaneous labor, complicating their ability to reach a health facility in time for delivery.	How-might we initiate shared decision-making between couples to encourage first-trimester ANC attendance? How-might-we support pregnant women and their husbands in making shared decisions about institutional delivery?
*Inconvenient Journey to and from a Health Facility:* Vulnerable pregnant women typically live far from health facilities, with challenging topography adding to the difficulty of travel. Motorcycles, the primary mode of transport in the two woredas of implementation, are inconvenient and uncomfortable for pregnant women, as they are usually crowded with multiple passengers and navigate bumpy, unsafe roads.	How-might-we improve the journey for pregnant women to and from health facilities to increase the likelihood of delivering there?
*Heavy Workload and Lack of Household Support*: Even in advanced gestational age of pregnancy, women bear a heavy workload in the house including childcare, cooking, and cleaning, without support from husbands or other family members. The absence of assistance increases stress and fatigue, leaving women less able to prioritize their own health needs.	How-might-we support husbands in prioritizing ANC within their households to encourage their wives’ attendance?
*Negative Perceptions of Public Health Services:* Many pregnant women are discouraged by perceived mistreatment at public health facilities. Women feel that visits to healthcare providers often yield minimal benefits as they frequently leave health centers without new or helpful information. This perception diminishes the perceived value of health services, deterring women from seeking regular ANC.	How-might-we improve access to information for low-literate community members about maternal and child health, encouraging early ANC attendance? How-might-we raise awareness and understanding of ANC benefits so that pregnant women prioritize it?
*Low Motivation and Poor Working Conditions for Health Service Providers:* Health service providers experience significant dissatisfaction due to low pay, burnout, and poor working conditions. Many providers live far from their workplaces and endure long, uncomfortable commutes. Additionally, health facilities often lack basic amenities, such as running water and reliable electricity, further straining providers’ morale and their capacity to deliver quality care.	How-might-we enhance service friendliness during delivery to encourage women to choose health centers for childbirth? How-might-we create a welcoming and friendly service environment to encourage pregnant women to seek early ANC?
*Limited Awareness and Access to Information:* Both pregnant women and their husbands lack adequate information about the benefits of accessing antenatal care and institutional delivery. While health extension workers encourage them to attend their antenatal care appointments and deliver at health facilities, pregnant women often find the journey too burdensome. Health posts, which are closer than health centers, offer some antenatal care services, but health extension workers are not consistently available at these locations, discouraging women from making even these shorter trips.	How-might-we increase pregnant women’s awareness about the importance of delivering at a health facility? How-might-we build pregnant women’s confidence in health facility services, so they opt for institutional delivery?
*Delayed Pregnancy Recognition:* Many pregnancies in the community are unplanned, and pregnant women often do not recognize early signs. This delay in recognizing pregnancy results in delayed engagement with antenatal care services. By the time women realize they are pregnant, they are typically further along in their pregnancies, reducing the likelihood of early antenatal care attendance and preparation.	How-might-we improve community support for pregnant women to start ANC within the first trimester? How-might-we enable HEWs to identify early pregnancies among women in remote areas, so they start attending ANC?

These insights and HMW statements provided a foundation for ideation, helping the design teams generate practical, community-centered ideas for addressing the identified challenges.

### Ideation and prioritization

Like the first-round of workshops, the ideation step was done in three sub steps: rapid individual brainstorming, followed by group brainstorming, and then prioritization of ideas. Furthermore, in the second round of workshops, between the first two sub steps before the group brainstorming started, the workshop facilitators jumbled up the individual ideas with prioritized ideas from the first round of workshop so that all ideas were considered in the group brainstorming and prioritization of ideas.

**Fig 6:**
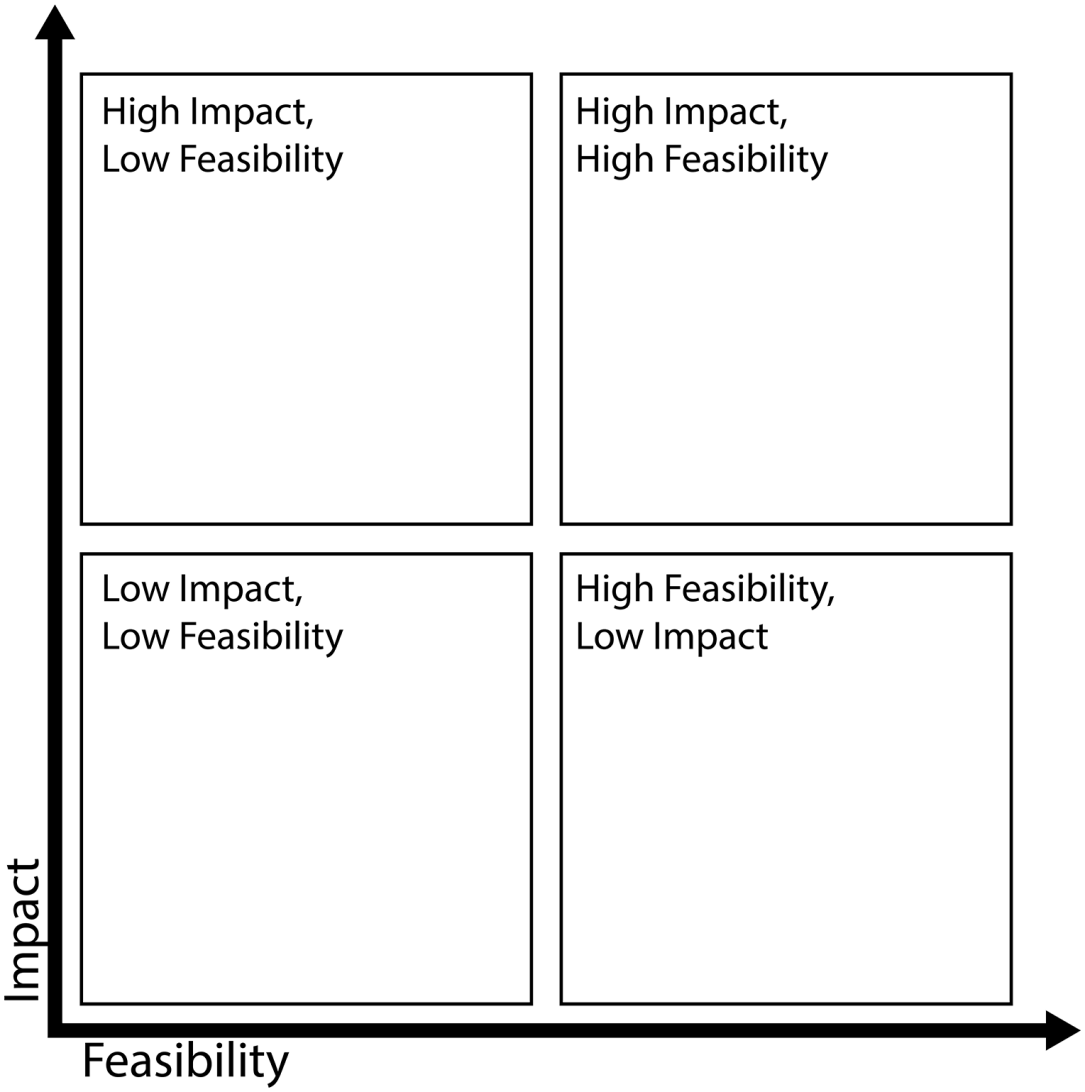
Impact-feasibility matrix used to prioritize ideas.

In the second round of workshops, participants were first encouraged to write ideas for the prioritized how-might-we statements individually. Facilitators took the individual ideas and presented them combined with ideas from the first round of workshops under the relevant how-might-we statements to ensure that all contributions were considered. A total of 62 initial ideas emerged from rounds of workshops, which were further prioritized using an impact and feasibility matrix. The initial set of ideas was refined through discussions and support from the facilitators. The most promising ideas were then prioritized, considering three main criteria—desirability, viability, feasibility—leading to the development and refinement of solutions to improve maternal health outcomes in the study areas. However, to make it easy for selection, participants used (x, y) coordinate graphs, where x represented feasibility and y combined viability and desirability as impact (shown in Fig 6). The participants debated among themselves before deciding on the impact and feasibility of each idea, leading ultimately to a consensus. After having a final set of prioritized ideas, the core team did further prioritization mainly from a feasibility point of view given the project scope and available resources using the same template (**Fig 6**).

### Prototyping

Several low-fidelity prototypes emerged from this process. Among these, four key prototypes were more fully developed as potential solutions.

1. *Alternative Transportation and Maternity Waiting Homes:* This prototype addressed the challenge of inaccessible health centers due to distance and inadequate transportation. The design team proposed utilizing locally made motorcycle extensions as an alternative means of transportation for pregnant women. These extensions could be kept at health centers for easy access. Additionally, improvements to existing maternity waiting homes—such as making them more comfortable and welcoming—were proposed to encourage institutional delivery.
2. *Family Conversation and Couple Discussions:* Recognizing the importance of family support, this prototype focused on fostering open communication between pregnant women and their husbands. The design involved providing couples with audio-based stories delivered through simple radio devices. These stories, focused on antenatal care and birth preparation, promoting institutional delivery, encouraged couples to pause, reflect, and discuss key aspects of pregnancy, health services, and supportive roles. Couples listened to the stories privately, fostering open dialogue and joint decisions about the importance of ANC and health facility delivery as the pregnancy progressed. This idea was later refined into the self-paced, audio-based dialogue model, with a visual print material that reinforced the contents of self-paced audio, which was added as the final prototype.
3. *Motivation for Service Providers:* Based on insights about service provider dissatisfaction, this prototype aimed to improve the workplace environment for midwives, health extension workers, and women development armies. Suggested improvements included creating cleaner, more comfortable delivery rooms, and organizing semi-annual recognition events to acknowledge the efforts of service providers. These changes were designed to improve provider morale, and, in turn, the quality of service offered to pregnant women.
4. *Early Pregnancy Screening:* To address the need for early antenatal care initiation, this prototype proposed proactive early pregnancy screening in remote areas. Health extension workers would first map households with women of reproductive age who were not using family planning. Through home visits, they would discuss pregnancy signs and provide home pregnancy test kits to women showing early signs. Women testing positive would be given referral cards to the health center. This prototype directly addressed the challenge of identifying pregnant women early, encouraging timely antenatal care attendance, which is a gateway behavior for institutional delivery.

Out of these ideas, the family conversation and couple discussion intervention was selected for further refinement, which resulted in the self-paced audio-based couples’ discussion prototype.

### Prototype testing and iteration

The prototypes underwent two testing rounds to assess feasibility, acceptability, and relevance to healthcare providers and couples. The first round involved 24 selected design team members from initial workshop participants, who validated the low-fidelity prototypes in a workshop setting to make sure that their ideas and suggestions were incorporated in the prototype. A low-fidelity prototype was presented to the design teams to get their feedback before the core team worked on a mid-fidelity prototype. The participants appreciated the prototype and suggested incorporating a guide for women development armies who were poised to play a crucial role in the implementation of the intervention. The core team incorporated their feedback and developed a mid-fidelity prototype that was more fully developed to allow people to interact with it more meaningfully.

The second-round prototype testing (testing of mid-fidelity prototype) was done with 36 participants of six focus group discussions (FGDs) that are different from the design teams (workshop participants from the first and second rounds of workshops). The intention was to gather independent views. They were purely recruited to try out the intervention (prototype) and provide feedback to improve it. Half of these were pregnant women who meet the same vulnerability criteria while the other half were husbands. A similar recruitment approach was made as the selection of the design team participants, with none declining to take part. A total of 87 households were assessed to identify the 36 participants in six FGDs. We have Annexed summary of the demographic characteristics of the FGD participants. Both of these activities (validating the low fidelity with design team members and testing the mid fidelity with new participants) were part of the testing step in the design thinking process. Participants interacted with the audio-based prototype over a period of several days (three days session of listing and discussion per couples), with health extension workers and women development armies facilitating distribution and usage instructions. Feedback was collected through focus group discussions, allowing participants to share their experiences and any challenges they encountered. Most responses were positive, with participants expressing appreciation for the relatable storytelling and the ease of engaging with the content in their own time. Minor suggestions were received, primarily regarding the clarity of some audio content. It was mainly a revision of the stories to fit with culture and local languages and specific discussions questions for after listening to the audio. Based on this feedback, minor adjustments were made to improve the prototype’s usability and appeal. Both testing took place in both Shalla and Siraro woredas to ensure that perspectives across different settings were captured.

### Final solutions

Through prototyping and testing phases, the final solution was a couple-centered intervention- self-paced audio-based content to encourage couple discussion about the importance of ANC visits and institutional deliveries. This content was uploaded onto a device and delivered to couples through women development army home visits.

This intervention was selected due to its broad applicability to both initial design challenges, strengthening support for antenatal care and promoting institutional delivery among vulnerable couples—and addressing multiple how-might-we statements. This prototype would strengthen the human-centered nature of the solution because couples’ preference and acceptance were checked during prototype testing phases. The intervention focused on fostering couples’ discussions pertaining to antenatal care visits and institutional delivery through a relatable, audio-based storytelling format, which participants could engage in privately at their convenience. A summary of the real-life stories **(**content of couples’ conversation) focusing on Antenatal care and institutional delivery is shown below.

### Key highlights from the Antenatal care (ANC) story

This audio starts with a narrator congratulating the couple on pregnancy. In order to break the ice and initiate the conversation between the couple, the narrator prompts them to pause the audio and tell each other how they have been doing through the pregnancy so far, and whether or not they have started ANC and how they feel about it.

Next the narrator introduces a couple who proceed to tell their story with their own voice. This couple, who met in school and are now both teachers, share their journey of marriage and starting a family. The wife, after moving for her husband, became pregnant shortly after their wedding. Motivated by the tragic loss of her aunt during childbirth, she worried about her own delivery, but her husband’s constant reassurance and support alleviated her fears. He actively participated in household chores and accompanied her to distant antenatal care appointments, enduring societal ridicule for his involvement. Their mutual agreement and the husband’s efforts, including reading a book to prepare for the baby, led to a safe delivery for the wife, who now happily encourages her friends to seek facility-based care during pregnancy.

Following the story, the narrator prompts the listeners with specific questions based on the story. These prompts are designed in such a way that the listeners, while relating to the story, can also reflect on their own journey through the pregnancy and their ANC experience. The questions also spark discussions between the couple on what they need to do to overcome challenges for ANC. The audio ends after the narrator outlines specific recommendations for the ANC uptake for the listening couple.

### Key highlights from the health facility delivery story

This audio starts with the narrator congratulating the couple on the pregnancy and encouraging them to do the “right” thing in the last days of the pregnancy. As an ice breaker, the narrator then asks the couple to pause the audio to tell each other about how they have been feeling. As the audio continues, the narrator introduces the storyteller who speaks of her own experience related to pregnancy and childbirth.

As the woman tells her story, she and her husband initially were oblivious to the signs of pregnancy, only discovering the truth through her mother’s discreet guidance, as such topics were considered too embarrassing to discuss openly. Societal norms encouraged pregnant women to work strenuously, believing it would ease labor, a notion even her own mother espoused, leading her to perform demanding tasks despite her growing fatigue and inadequate nutrition.

As her delivery date approached, she found herself in a prolonged and agonizing labor that lasted a week. Surrounded by well-meaning neighbors who offered traditional remedies and shifted her position, she felt a profound sense of hopelessness and fear, convinced she would die. Her cries were suppressed, and she was confined indoors to prevent others from hearing her pain. Despite her immense suffering, she was repeatedly told that childbirth was inherently painful and that her struggles were normal. The story ends with her and her community deeply worried as her arduous labor continued.

At this cliffhanger, the story pauses and the narrator asks the listeners to discuss with each other on what the woman and her husband should have done to prevent or put an end to the suffering. After this, the woman continues to tell her story. She recounts how she finally gave birth after a week-long, traumatic ordeal that nearly cost her life due to a home birth. For her second pregnancy, thanks to the guidance of Health Extension Workers, she was well-prepared and went to a healthcare facility. This experience was quick and positive, highlighting the life-saving difference professional medical care made. She now advocates for facility-based births, urging others to learn from her near-fatal home birth.

Based on this story, the narrator prompts the couple listening to the story to reflect on their current pregnancy, and about what they need to do. The narrator finally outlines the benefits of institutional delivery and service uptake before the audio ends.

The audio content was translated into Afan Oromo, the local language and recorded into radio devices containing the audio with appealing classical sounds. Furthermore, print materials that were intended to reinforce the audio content were developed focusing on antenatal care visits and institutional delivery. The print materials were an add on messages on ANC and health facility delivery designed for rural illiterates with visual illustrations to reinforce the audio contents that were translated into local language for easy understanding. These print materials could help couples and family members to understand the issue of ANC and health facility delivery. Home to home visits by women development armies besides to rotating audio devices and deliver print materials could be also part of our solutions in mobilize community- and system-level resources for scalability. The resulting intervention was part of an implementation research project aimed to develop and test solutions that optimize antenatal care and institutional delivery among vulnerable pregnant women in Ethiopia.

### Implementation

The interventions (solutions) that informed by HCD were implemented using the existing community health structure, specifically health extension workers, and women development armies. The implementation followed a structured process to ensure accessibility, ease of use, and effectiveness in reaching households across two woredas, Shalla and Siraro. An adaptive implementation strategy was applied with seven components included as described below.

***Training and Preparation***: Health extension workers received training on the intervention and were provided with audio devices and memory sticks containing ANC-related stories. The audio content was designed to guide couples through important discussions about maternal health, promoting shared decision-making and supportive roles during pregnancy. Health extension workers also received supplementary print materials to enhance engagement with the content.

***Orientation of women development Armies (WDAs)*** Health extension workers collaborated with women development armies, orienting them on how to use the audio devices and content effectively. Women development armies were introduced to the content’s objectives and the proper handling of the supplementary print materials, which included visual prompts to aid discussion within households.

***Identifying and Engaging Vulnerable Households***: Women development armies identified households with pregnant women who were beyond 5 months of gestational age and who met the vulnerability criteria. An initial appointment was scheduled with each household, involving the couple and other available family members.

***Initial Meeting with Couples***: During the first meeting, women development armies used print materials to discuss ANC and its importance, creating a foundation for the upcoming audio-based discussions. They instructed couples on how to operate the audio device and explained that they could listen to the content privately at a convenient time. Women development armies left the audio device, memory stick, and ANC-related print materials with the couple, ensuring they had resources to revisit key information.

***Couple Dialogue Using the Audio Content***: Couples listened to the audio stories at their convenience, guided by prompts within the audio to pause, reflect, and discussed specific questions related to their pregnancy journey. The self-paced format encouraged open communication, with each story presenting relatable scenarios to enhance the couple’s understanding of ANC and build a supportive partnership. The audio stories 1 and 2 were packaged separately and given to the couples one after the other. The first one was given when the women are 5–7 months of pregnancy while the second story was given to them when they are 8–9 months pregnant. There were print guides and messages that were given along with the audio stories. The WDAs also conducted discussions with the pregnant women, their husbands and other available family members giving them instructions on how to use the audio devices and going over the accompanying with visual print material.

***Follow-Up and Audio Device Rotation***: After a few days, the women development army leader returned to follow up with the couple, collecting feedback on their experience and addressing any questions. The audio device was then retrieved to be shared with another identified couple, allowing the intervention to reach multiple households over time.

**Fig 7:**
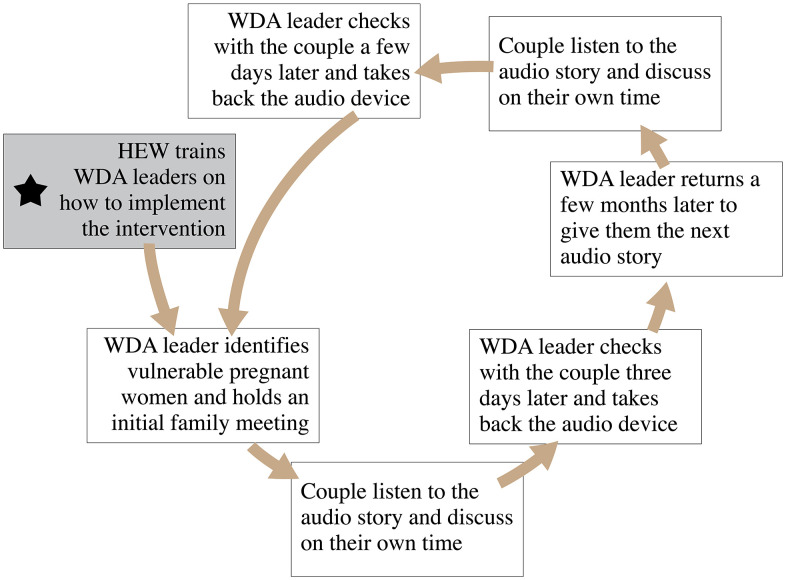
Implementation process to support community workers during implementation at larger scale.

***ANC, Birth Preparedness and Health Facility Delivery Sessions:*** As the couple approached the final trimester, the women development army leader returned for a second session, this time focused on birth preparedness. The process was similar, with a new audio story and supplemental print materials emphasizing the importance of preparing for institutional delivery. This additional session reinforced the couple’s commitment to institutional delivery and prepared them for a safe childbirth experience. The implementation process is summarized below to show how the intervention (prototype) reached to households with vulnerable pregnant women (**Fig 7**).

By integrating into Ethiopia’s established public health network and leveraging the existing trust in health extension workers and women development armies, the intervention was able to effectively engage vulnerable couples, promoting ANC and institutional delivery in 1127 households that meet the vulnerability criteria. The implementation approach allowed participants to interact with the content at their own pace, fostering a supportive environment for maternal health discussions.

## Discussion

The main objective of our design thinking process was to co-create feasible and effective solutions to improve antenatal care visits and institutional delivery among vulnerable pregnant women who are underserved by routine maternal and child health services. Our design thinking process helped us uncover insights into the specific challenges of vulnerable women in accessing maternal health services. By involving pregnant women, their husbands, and key stakeholders in the design process, we were able to identify relevant needs and develop a feasible, culturally appropriate prototype, though the effectiveness of this effort, from the perspective of improving ANC visits and institutional delivery, has yet to be ascertained.

The self-paced, audio-based couples’ dialogue was designed to encourage open conversations between couples about antenatal care visits, birth preparedness, and health facility delivery. By directly addressing participants’ concern about the lack of shared decision-making and responsibilities within households, our intervention attempted to empower women through supportive relationship dynamics. This approach was hypothesized to be effective in the setting where inequitable gender norms and conventional male roles prevail, involving husbands and incorporating messages emphasizing male roles in maternal care to create a supportive environment for vulnerable pregnant women. This aligns with other studies that highlight the importance of family and partner support in improving ANC attendance and institutional delivery [29,30]. Also, a qualitative study in Malawi showed that rural women preferred family planning interventions promoting spousal communication and shared decision-making to clinic-based education where women often participate alone [27]. Despite the potential of couple-based behavior change interventions, we acknowledge the need to explore whether this approach will work in other areas with different cultural and social values and norms.

Our study also highlights some of the practical advantages of using relatable storytelling in healthcare interventions. The audio-based format, which required minimal literacy skills, allowed couples to reflect on relatable scenarios at their own pace. This approach has been shown to improve health service seeking and utilization in previous studies, as it respects user autonomy and addresses accessibility barriers [22]. Yet, given that most households were not equipped with radio devices, our implementation model required securing radio devices and involving designated personnel to manage and rotate the devices in the community. While this was considered the most feasible delivery mechanism in our intervention areas, the issue of lost or damaged devices and contextual barriers hampering radio rotation was reported. However, some of these limitations were overcome by modifying our implementation strategies, such as involving community volunteers who can provide additional support with radio rotation. Our approach to mobilizing community networks and established platforms in the health system enhanced feasibility, aligning with Roberts et al.[31] findings on the effectiveness of HCD interventions within local health systems.

While the self-paced, audio-based couples’ dialogue was prioritized for its feasibility, other prototypes that emerged during our design thinking process could have addressed significant challenges as well. Ideas such as alternative transportation solutions, improvements to maternity waiting homes, and early pregnancy screening were identified as essential to improving maternal health outcomes. These additional solutions, if implemented, may provide further support for vulnerable women, addressing both logistical and structural barriers to facility-based deliveries. Such multi-faceted approaches are essential in addressing complex health challenges such as maternal mortality, as documented in studies on community-driven health initiatives [29,30].

The iterative nature of our design thinking approach allowed for refinement of solutions by end-users and relevant stakeholders. This participatory approach builds on existing literature that underscores the importance of tailoring health interventions to the needs and preferences of diverse community members [31]. However, ensuring the sustainability of the intervention will require ongoing community engagement and potential adaptation to suit other cultural or geographic contexts [32]. As highlighted in prior HCD studies, iterative refinements of solutions and other design principles, such as sustaining community stakeholder engagement and training design practitioners and researchers, are time and resource intensive [33,34]. We thus recommend allocating a sufficient timeline and budget in planning design-based programs.

### Program implications

This study demonstrates the potential of HCD to create targeted, culturally sensitive, human-centered interventions that can be integrated into existing community health structures. We also engaged key stakeholders, such as end-users, the health center directors and woreda maternal and child health leaders throughout the design process. We also solicited the active involvement of health leaders while the proposed solutions were being developed, with the assumption that doing so would make for a more effective program and also increase their willingness to implement and sustain interventions within the community. Successful sustained implementation of the prototypes we developed would ultimately depend on buy-in from local health leaders [32].

The intervention required significant time investment over four months, allowing for thorough community engagement, but this period was critical in ensuring that solutions were well-tailored to local needs. The initial success of the audio-based, self-paced couples’ discussion intervention, which was implemented in the two woredas in the Oromia region, suggests that HCD can be applied in similar contexts to improve maternal health outcomes among vulnerable populations. While the implementation phase is ongoing, continuous iteration and adaptation of the intervention in response to user feedback will further support its long-term viability. Collaboration with local government health systems will also be crucial in scaling up the intervention to ensure its accessibility to all pregnant women within the community, facilitating its integration into the broader maternal and child health framework.

The evaluation findings of the HCD intervention will be analyzed, summarized, and reported, as outlined in our study protocol [35]. The implementation rollout and subsequent evaluation of the prototype is ongoing and continues to be iterated in response to user feedback. For our work to make family conversation and couple discussion through recorded stories to be accessible to all pregnant women, we are working closely with local government health system in our community to better integrate their doulas and antenatal care health workers into the broader team, and to create scalable and sustainable model.

### Strengths and limitations

A key strength of this study lies in its iterative, human-centered approach, which actively engaged vulnerable pregnant women, their partners, and local health stakeholders. This participatory method ensured that the intervention closely aligned with the needs and preferences of its users, enhancing both its cultural relevance and acceptability. Furthermore, the resulting solutions of simple audio-based storytelling and visual print materials made the intervention accessible to low-literacy households, increasing its usability among the target population. By integrating the prototypes within the existing public health structure, specifically through health extension workers and women development armies, the study leveraged established community trust and resources, which supported a feasible and scalable implementation.

Additionally, audio recordings and direct transcriptions during workshops were deliberately avoided to respect participant confidentiality, which may have affected the accuracy and reliability of our HCD findings. Lastly, while the design thinking teams included vulnerable pregnant women, husbands, and health workers, these groups were separately engaged to maintain homogeneity and manage groups dynamics. This approach, however, may have limited the ability to capture the full range of perspectives across the broader population. Our prototype solutions may not be broadly generalizable across the wider geographical context, given the country’s extensive size and the diverse socio-economic and cultural diversities, particularly among vulnerable pregnant women. Nonetheless, the use of iterative feedback loops across multiple design thinking phases, involving brainstorming and synthesis, likely reinforced the validity of core findings.

## Conclusion

This study demonstrates the potential of HCD in addressing barriers to maternal health service use among vulnerable pregnant women in Ethiopia. Our work highlights the value of a design thinking approach in creating community-tailored, accessible health interventions. By engaging both direct beneficiaries and health system actors, we developed an innovative, scalable prototype that has the potential to improve antenatal care, birth preparedness and institutional delivery rates among vulnerable women in Ethiopia. Future studies should explore the long-term impact and scalability of this intervention, along with additional prototypes, to better address maternal health challenges in underserved communities.

## Supporting information

S1 Table Demographic profile of design team and prototype testing participants. (DOCX)

S2 Table Operational definition of key terminologies that we used throughout our HCD process. (DOCX)

S3 Table Real life stories focusing on Antenatal care and institutional delivery. (DOCX)

S1 File Checkilst. (DOCX)
