# Supplementary material for: Transforming maternal health in Ethiopia: Leveraging human-centered design to co-create innovative behavioral interventions
Source: PLOS Glob Public Health. 2026 Feb 24;6(2):e0006021. doi: 10.1371/journal.pgph.0006021 (PMC12931744; doi:10.1371/journal.pgph.0006021)
Supplement: S1 Table — (DOCX) [file pgph.0006021.s001.docx]

**S1 Table:** Demographic profile of design team and prototype testing participants

| **Round 1 Workshop Participants profile** (PW and Husbands, n=96) | # of participants |
| --- | --- |
| - Pregnant women with vulnerabilities | 48 |
| - Husbands of pregnant women | 48 |
| - Women age range 18-49 | 48 |
| - Husbands age range 18-49 | 48 |
| - Women with no formal education | 34 |
| - Women with formal education | 14 |
| - Husbands with no formal education | 8 |
| - Husbands with formal education | 40 |
| - Rural pregnant women | 36 |
| - Urban pregnant women | 12 |
| - Rural husbands | 32 |
| - Urban husbands | 16 |
| **Round 2 Workship Participants profile** (Healthcare providers, n=48) |  |
| - Health extension workers (HEWs) | 8 |
| - Midwives/nurses | 4 |
| - Primary Health Care Unit (PHCU) directors or deputies | 6 |
| - Women Development Armies (WDAs) | 6 |
| - Local actors (religion leaders, women affair, kebele leaders) | 20 |
| - Maternal and Child Health staff from the worker’s health office | 4 |
| - Male participants | 33 |
| - Female participants | 15 |
| - Local actors with no formal education | 2 |
| - Local actors with formal education | 46 |
| - Age range 18-49 | 48 |
| **Round 1 Prototype Testing Participants profile**, n=30, 2 workshops |  |
| - Pregnant women with vulnerabilities | 3 |
| - Husbands of pregnant women | 3 |
| - Health extension workers (HEWs) | 8 |
| - Midwives/nurses | 4 |
| - Primary Health Care Unit (PHCU) directors or deputies | 2 |
| - Women Development Armies (WDAs) | 3 |
| - Local influencers (religion leaders, women affair, kebele leaders) | 5 |
| - Maternal and Child Health staff from the worker’s health office | 2 |
| - Male participants | 11 |
| - Female participants | 13 |
| - Have no formal education | 3 |
| - Have formal education | 21 |
| - Age range 18-49 | 24 |
| **Round 2 Prototype Testing Participants profile**, n=36, 4 FGDs |  |
| - Pregnant women with vulnerabilities | 24 |
| - Husbands of pregnant women | 12 |
| - Male participants | 12 |
| - Female participants | 24 |
| - Have no formal education | 19 |
| - Have formal education | 17 |
| - Age range 18-49 | 36 |
