# Supplementary material for: Transforming maternal health in Ethiopia: Leveraging human-centered design to co-create innovative behavioral interventions
Source: PLOS Glob Public Health. 2026 Feb 24;6(2):e0006021. doi: 10.1371/journal.pgph.0006021 (PMC12931744; doi:10.1371/journal.pgph.0006021)
Supplement: S2 Table — (DOCX) [file pgph.0006021.s002.docx]

**S2 Table: Operational definition of key terminologies that we used throughout our HCD process.**

| **Terms** | **Short description/definition** |
| --- | --- |
| Design challenge | The specific problem or opportunity of focus for the HCD approach. It is formulated in a way that is open-ended enough to inspire diverse solutions, while still being focused enough to guide the problem-solving process. |
| Design thinking | A transdisciplinary, human-centered, creative, problem-solving approach that emphasizes empathy, perspective-taking, iterative prototyping, and testing. The design thinking process includes five distinct, yet interconnected, steps—empathize, define, ideate, prototype, and test. |
| Co-design/creation | An approach to design thinking that emphasizes creative collaboration between stakeholders to produce innovative ideas and solutions. |
| Stakeholders | Anyone related to the design challenge of focus, regardless of degree of relationship. Stakeholders are commonly engaged to varying degrees depending on their specific stake in the challenge of focus. |
| Empathy | Empathy is the foundation of a human-centered design process; by deeply understanding people we are better able to design for them. |
| Empathize Phase | Learning about and from the stakeholders for whom you are designing |
| Define Phase | Identifying the positives, challenges, and opportunities related to the design challenge and nothing key themes and insights |
| Insights | Insights are ideas or anecdotes expressed as succinct statements that serve to interpret patterns in research findings. Insights offer a new perspective, even if they are not new discoveries. They are inspiring and relevant to the design challenge. |
| Affinity clustering | The process of grouping similar data points together in order to identify key themes and insights. |
| How Might We Statements… | An open-ended question that frames a design challenge in a way that encourages innovative thinking and problem-solving. It is the springboard for research, design, ideation, and iteration. |
| Ideate Phase | Brainstorming creative solutions to the challenge of focus, rooted in the insights from the empathize and define phases |
| Prototype | A tangible or visual representation of the solution(s) from the ideation phase. Prototypes range from low to high fidelity and are intended to be tools for feedback and iteration. Examples of prototypes: skit, model, storyboard, simulation, pop-up, video, diorama |
| Prototyping Phase | Building a tangible or visible representation of one or more of the proposed solutions from ideation for testing |
| Persona | It is a representation of a user segment with shared needs and characteristics. In HCD, personas are archetypal characters that represent different user segments that might engage a product or service in a similar way. Sometimes referred to as an archetype |
| Journey map | A journey map is a framework that can help designers think through and strategize about key moments for different stakeholders as they go through a certain journey (general or specific to a proposed solution). A journey map can, for example, lay out: how stakeholders first become aware of a solution; what their initial interactions and engagement with it are like; how they might become a repeat user; and how the solution might ultimately impact their life. |
| Iteration | Iteration is the act of testing and refining ideas with the aim of approaching a desired goal. Each repetition of the process is called an iteration. In design, it refers to the cycles of learning, creating, prototyping, and measuring. Designers typically go through several rounds of iteration in which they present their ideas and prototypes to users and then make incremental changes based on their feedback. This process leads to ideas that are more in tune with user needs. |
| Feasibility/Impact Matrix | A tool that helps prioritize tasks and problems by categorizing them based on their importance and difficulty or feasibility and impact. It's a design thinking framework that can help with: Prioritizing: Quickly prioritize tasks and items, understanding: Understand the relative difficulty of tasks, collaborating: Get broad team alignment on action items, Planning: Develop a plan of action. |
| Vulnerability | Vulnerability refers to underserved pregnant women affected by diverse social, environmental, and behavioral factors. |
| Hadha Garee | Hadha Garee is an Affan Oromo local term to describe women development army who promote health for nearly 30 pregnant women in her village. |
