# Supplementary material for: Transforming maternal health in Ethiopia: Leveraging human-centered design to co-create innovative behavioral interventions
Source: PLOS Glob Public Health. 2026 Feb 24;6(2):e0006021. doi: 10.1371/journal.pgph.0006021 (PMC12931744; doi:10.1371/journal.pgph.0006021)
Supplement: S3 Table — (DOCX) [file pgph.0006021.s003.docx]

**S3 Table: Real life stories focusing on Antenatal care and institutional delivery**

| **Audio contents of antenatal care (ANC)** | **Audio contents of institutional delivery (ID)** |
| --- | --- |
| **Section 1:**  Congratulations on your pregnancy. We know you will be amazing parents for this child. It is important that the two of you support each other, openly discuss and do what is right for the health of the child and the family.  Before we continue, please pause this audio and talk to each other for a bit. Please let your husband know how you are feeling during this pregnancy physically and emotionally. What makes you happy and what worries you? If you have started going to ANC, please share how your experiences have been. If not, talk about why you haven’t done so until now.  Please pause the audio now and discuss it. When you finish, hit the play button and listen to the rest of the audio. | **Section 1**:  You are just a few weeks before you become new parents. Congratulations! We know you will be amazing parents for this child. It is important that the two of you support each other, openly discuss and do what is right for the health of the child and the family.  Before we continue, please pause this audio and talk to each other for a bit. Please let your husband know how you are feeling during this pregnancy physically and emotionally. What makes you happy and what worries you? What preparations have you made so far to receive your new baby?  Please pause the audio now and discuss it. When you finish, hit the play button and listen to the rest of the audio. |
| **Section 2:**  We will hear the story of a couple that gave birth to their first child some time ago. These are real people just like you, and they were gracious to share their story with us. The woman you will hear is [name of the woman included] and her husband is [name of the husband included]. They met when they were in school, and they are now both teachers. Let’s listen to their story.  ***Husband****:* We decided to get married soon after she finished school  ***Wife****:* He loved me so much. So, I left my family and my friends and came to this area for him. Not too long after, maybe about two months later, I became pregnant.  My aunt’s husband never supported her when she was pregnant. She did everything by herself – cutting wood, fetching water, carrying loads on her back, everything. And then I heard she passed away during labor. It was very sad. So, when I think of her, I used to worry if I would make it. But whenever I talked to him, he made me feel better. He used to tell me not to worry and that nothing would happen to me.  ***Husband****:* I tried to do everything I could to help her when she was pregnant. Even then, I used to worry a lot thinking about what might happen. But then when I thought that we had already started going to the facility for ANC and that she would be delivering in the health facility, I felt better.  When she was pregnant, I used to do a lot to help including house chores like washing the dishes, making the bed, fetching water, cutting firewood, and everything. But there were people who mocked me for doing those things.  At the time, the health center was very far, about 13 kilometers. We used to go there to complete the four rounds of visits when she was pregnant to consult with the doctor. I did everything I could to make sure she and the baby were healthy. It was not easy. People talked behind my back saying, “Why is he, as a man, going to the health center with her? Why doesn’t she go there by herself?”. I ignored them. I wanted both to be healthy, so I went with her to the facility.  ***Wife:*** They used to say, “she makes her husband work. Her husband has become submissive and has become a house pet. They shame men who supports with household chores. But he and I had an agreement, so we didn’t mind them. There was a book that he read and shared with me about what to do for me and the baby. It used to make me very happy.  Alhamdulillah! I had a safe delivery. Now I have two kids. And I feel very peaceful. I am happy. I advise all my friends to go to the health center when they are pregnant.  ***Husband:*** A couple needs to look out for each other. When she is pregnant, he is also pregnant. He has to do his part. He has to go with her to the health facility and help in the house.  We hope you have enjoyed the story of the couple. Please share with each other how the story made you feel. Talk about your own experience in light of the story. What support do you need from one another? When are you planning to go to the health facility next for ANC? What preparations do you think you need to make for the birth of your baby? What are some of your challenges and what can you do about them?  Please pause the audio now and discuss it. When you finish, hit the play button and listen to the rest of the audio.  **Section 3:**  We hope you have had a good time, and you have got to reflect on what you should be doing to protect the health of your child and your family. It is crucial that you start attending ANC at the health facility if you have not done so. ANC will help you identify and treat any complications early on in pregnancy, which can improve the health outcomes for both the mother and the baby. It also helps you plan better for delivering in a health facility with health professionals who can help you safely deliver your baby.  There are plenty of things that could go wrong during delivery that may cause serious threats to the mother or the baby. The only way you can avoid these threats is if you have a trained professional with the necessary equipment with you when you deliver.  The husband must do his part to make sure the wife visits ANC. The husband must accompany the wife when she visits ANC. Prepare for the trip and transportation to go to nearby health facilities. Take care of the wife and help with house chores.  We hope this has been enjoyable for you. Please continue to discuss openly with one another and support each other. Discuss when your next ANC visit will be and what you would need to go to the health facility. We hope to see you in another episode soon. Until then, keep talking to each other openly about how you feel, what your experience going to ANC and how you can make it better.  Please keep this device safe and return it to the Hadha Garee who gave it to you when she comes to collect it from you. | **Section 2:**  We will hear a story from a woman who had had a child not long ago. Her name is Hawa, and her story is about something we can learn from to keep us and our new baby healthy. Let’s listen to her.  My name is *Hawa Ahmed*. When I got sick and felt nauseated, I did not know what it was. And my husband also thought it was just some illness and didn’t know what it was. It was my mother who told us pregnancy has these symptoms. It was such an embarrassing topic that we didn’t talk about such things. You can’t even tell your mother about it. In any case, we eventually figured out it was pregnancy.  Working during pregnancy is often seen as something good. People say so and so’s wife is very brave because she works hard when she’s pregnant. So, they encouraged her to work even harder. Nobody would advise a pregnant woman to take it easy, that hard labor can be damaging for the fetus. People believe working hard would make it easy when labor kicks in. Even my own mother told me carrying heavy loads is good for a pregnant woman. Since we often hear this, I did everything myself.  As I drew near my delivery date, I was very tired. I haven’t had enough food too around that time. Labor was very difficult. I couldn’t push hard. It was very difficult. Everyone from the neighborhood came together. Some hold me from my left, others from my right. I hear them say somebody’s hand is better so others would take turns holding me. Or they say so and so it is better to let her sit on that person. They toss me from one person to the other. They make me change positions hoping that whatever is holding up the baby inside my womb would let go.  I lost all hope. I believed I was going to die. When I say this, they tell me no. They say everyone goes through this. They tell me to stay quiet. They also didn’t allow me to go outside so that people would not hear my cries. I try to hold it in but when it gets too much, I just go out anyway. Then they would tell me to go back into the house.  I knew I was going to die but they told me not to worry and that giving birth is always painful. It went on like this for a whole week with people all around me. Some carry Jebena on their heads and pray; others carry stools. Some of them tell me to change the way I sit, and they move me from one place to another. I was extremely worried and so were they.  Hawa’s story is very emotional. What do you think will happen next? Could Hawa have avoided such an experience? How? Please pause the audio and reflect on the story. Tell each other how the story made you feel. When you finish, hit the play button and listen to the rest of Hawa’s story.  **Section 3**  After a whole week, I gave birth. It was such a traumatic experience.  [space]  When I gave birth to my second child, Health Extension Workers came to us to see how we were doing. When labor came, I was well prepared. I packed up my clothes and had people call the ambulance for me. I went there and the doctors welcomed me. It didn’t take long before I delivered my second child. The health professionals really helped. It was a realization for me that I would have suffered for the whole week if I hadn’t gone there. The health worker told me never to give birth at home. The next day, I went back home. I almost died the first time I was in labor. Now I tell everyone to learn from my experience.  How was Hawa’s second pregnancy experience? After hearing Hawa’s story, what preparations do you think you need to deliver your baby to a health facility? What challenges are you expecting in going to health facilities and how would you resolve the challenges? What can husbands do to help their wives deliver a baby in health facilities?  Please pause the audio now and discuss it. When you finish, hit the play button and listen to the rest of the audio.  **Section 4:**  We hope you have had a good time, and you had a chance to reflect on what you should be doing to protect the health of your child and your family. As we can learn from Hawa’s story, delivering in a health facility is not a matter of choice for the health of a mother and her new baby. Luckily, Hawa lived to tell her story. For many mothers, this is not the case. So, you need to act now and start preparing to deliver to a health facility.  The first thing you need to do is for the two of you to talk openly and discuss how you can support each other. This is the time that your wife needs your support so ask her what she needs and give her the support that she needs.  Decide which health center is closest to you to go. If there is an ambulance in the health center, make sure you have the phone number of the ambulance driver so that he can come and collect you when you are in labor. If not, arrange other means of transportation. It’s important that you save enough money to cover transportation costs or to cover other needs that may arise.  Make sure also that you prepare and pack all the clothing you need for yourself and your new baby. You will need clean clothes that you can wear before and after giving birth, both for you and the baby. Also prepare a comfortable space for the baby and the mother to sleep after giving birth.  The husband must actively support the wife during pregnancy. He can help her with minimal household chores because pregnant women should take enough rest. The husband may search and arrange the transportation beforehand so that the wife can travel to health facilities for delivery without a problem. The husband can also check with his wife to make sure everything is packed before traveling to the health facility.  Please continue to discuss openly with one another and support each other. If you have any questions, please ask the Hadha Garee or Health Extension Worker. They will be happy to help you.  We wish you all the best.  Please keep this device safe and return it to the Hadha Garee who gave it to you when she comes to collect it from you. |
